# Attitudes and beliefs of nurses and physicians about managing sexual health in primary care: A multi‐site cross‐sectional comparative study

**DOI:** 10.1002/nop2.641

**Published:** 2020-10-20

**Authors:** Juan M. Leyva‐Moral, Mariela Aguayo‐Gonzalez, Patrick A. Palmieri, Genesis Guevara‐Vasquez, Nina Granel‐Grimenez, Artur Dalfó‐Pibernat

**Affiliations:** ^1^ Department d'Infermeria Universitat Autonoma de Barcelona Facultat de Medicina Barcelona Spain; ^2^ Grupo de Investigación Enfermera en Vulnerabilidad y Salud (GRIVIS) Universitat Autònoma de Barcelona Barcelona Spain; ^3^ Nursing Research Group EBHC South America: A Joanna Briggs Institute Affiliated Group Lima Perú; ^4^ Center for Global Nursing Texas Woman's University Houston TX USA; ^5^ Office of the Vice Chancellor for Research Universidad Norbert Wiener Lima Peru; ^6^ College of Graduate Health Studies A. T. Still University Kirksville MO USA; ^7^ Department of Research Hospital Regional Lambayeque Chiclayo Perú; ^8^ Primary Care Center Horta Institut Català de la Salut Barcelona Spain

**Keywords:** attitude of health personnel, community health nursing, nurse practitioners, nurse–patient relations, physician–patient relations, primary care physicians, primary health care, sexual health, Sexualidad en Atención Primaria Questionnaire, surveys and questionnaires

## Abstract

**Aim:**

To understand the attitudes and beliefs of nurses and physicians about managing the sexual health of patients during office visits in primary care centres.

**Design:**

A questionnaire‐based, cross‐sectional multi‐centre study.

**Methods:**

The study was performed in 15 primary care centres in Barcelona (Spain), from December 2017–February 2018. Obtained data were analysed with descriptive and bivariate statistics.

**Results:**

Nearly half the participants believed they should manage sexual health in primary care, but a third of them disagreed this is a priority. Participants also believed patients are not comfortable speaking with them about sex. Statistically significant differences were observed between the professions as nurses more often reported receiving sexual health questions from patients and believed they had enough knowledge to appropriately respond. Most participants wanted additional education to speak with patients more comfortably and confidently about sex.

## 1 INTRODUCTION

Sexual health is generally defined as a state of physical, mental, and social well‐being in relation to sexuality (World Health Organization, 2015). This requires a positive and respectful approach to sexuality and sexual relations, as well as the possibility of having safe sexual experiences without coercion, discrimination and/or violence (Edwards & Coleman, 2004). Discussions about sexual health during a primary care visit should be approached like any other topic focused on health promotion. Yet, clinical professionals such as nurses and physicians report difficulties speaking with patients about sexual health due to the multifactorial and multicultural context (Barragan, 1995; Gott et al., 2004). In addition, stereotypes and myths about sexual topics persist despite decades of reforms in health science curriculums (Bayer et al., 2017; Frenk et al., 2010; Kong et al., 2009; Saunamäki & Engström, 2014; Stevens, 2013) that should have contributed to a more contemporary and professional approach with modern social practices (Nair & Webster, 2010). There has been little change in the way nurses and physicians address sexual health, from older patients (Leyva‐Moral, 2008) to pregnant women (O'Malley et al., 2015), during primary care visits.

## 2 BACKGROUND

In the primary care setting, patients present with conditions commonly reported to have a negative impact on sexual health including, depression and anxiety (Tijeras‐Úbeda, 2010), hypertension (Latif et al., 2014), menopause (Thornton et al., 2015), and diabetes (Bal et al., 2015). However, clinicians report negative experiences when managing sexual health problems, especially when the purpose of the visit is not perceived to have a direct relationship to sexual health (Fuzzell et al., 2016; Magnan & Norris, 2008; Magnan et al., 2005; Rashidian et al., 2016). For example, only 8% of American adolescents (*N* = 253) were able to initiate a conversation with their physician, while the remaining were either embarrassed to share information or they were fearful about judgement with unwelcomed comments (Alexander et al., 2014). The same authors also reported 65% of the adolescents received consultation or content related to sexual health during their visit, but the mean time was 36 s. Other studies have similarly reported sexual health conversations during primary care visits are low frequency with minimal depth (Donaldson et al., 2013; Klein & Wilson, 2002). Although sexual health is a historically important area for evidence‐based practice (Reynolds & Magnan, 2005), sexual health is often neglected by nurses and physicians (East & Hutchinson, 2013; Fennell & Grant, 2019; Tsimtsiou et al., 2006). Initiating sexual health discussions with patients has been described as opening a “can of worms” by Gott et al. (2004) due to the sensitivity, complexity, limited expertise and time constraints.

In Spain, little is known about sexual health in primary care. In a survey study of Spanish men (*N* = 1,500), 80% reported no experiences speaking about sexual health with their physicians. From the 20% of men reporting experiences, most were unable to understand their physicians because theyused incomprehensible terminology about sexual matters (Asociación Española para la Salud Sexual, 2011). Likewise, these beliefs and behaviours were reported to prevent sexual health conversations in another small study of Spanish nurses (Dalfó‐Pibernat et al., 2015). Understanding the attitudes and beliefs of primary care nurses and physicians about managing the sexual health of patients is necessary to tailor interventions that canincrease their engagement in the affective‐sexual education noted to have a positive impact on patient outcomes (Denno et al., 2015). The purpose of this study was to understand the sexual health attitudes and beliefs of Spanish nurses and physicians working in primary care centres in Barcelona (Spain).

## 3 THE STUDY

### 3.1 Design

This was a cross‐sectional multi‐site study with a questionnaire.

### 3.2 Participants

A purposive sample of nurses and physicians was recruited from primary care centres (PCCs) in Western Barcelona (Spain). From the 18 PCCs in this region, 15 (83.3%) agreed provide access to their staff for this study. These PCCs represent the diversity of the city. Family physicians, paediatricians, nurses and interns/residents (physicians and nurses) working in any of the PCCs were invited to participate. Only nurses and physicians not working in primary care, including those with temporary incapacity for work, were excluded from participation. An a priori power analysis was conducted to determine the proper sample size (*N* = 180) for the available population (*N* = 400), considering a 5% margin of error and a 95% confidence level.

### 3.3 Data collection

Data were collected electronically between December 2017–February 2018 using a questionnaire. Each PCC was sent a standardized email for their staff with the study information provided in a digital link to the questionnaire. The questionnaire was completed through a secure platform, Encuestafacil^®^, with submission confirming acceptance to participate.

#### 3.3.1 Questionnaire

The Sexualidad en Atención Primaria (SEX‐AP), or Sexual Health in Primary Care, questionnaire has 35 items, including 12 related to sociodemographics, 12 related to attitudes and 11 related to beliefs, with good reliability. The items in each domain were assessed with a five‐point Likert scale (strongly disagree, somewhat disagree, neither disagree nor agree, somewhat agree and strongly agree). The Spanish version of the questionnaire is presented in File S1, and the English version is presented in File S2.

### 3.4 Ethical considerations

After approval by the institutional ethics committee (# P17/230), the study protocol was presented to the PCC leaders for approval. Participation in this study was voluntary and without incentives. The data were managed with confidentiality, and no issues were reported.

### 3.5 Data analysis

At the conclusion of data collection, the data were downloaded into a Microsoft Excel (version 2010) spreadsheet to identify missing data and to organize the data for statistical analysis. Any questionnaire with more than a 20% non‐response rate (Dillman, 2007), or seven missing responses, was eliminated from analysis. No data imputation techniques were used. The statistical analysis was performed with the STATA (version 14). As the quantitative data may not be normally distributed, Kolmogorov–Smirnov test (Gravetter & Wallnau, 2016) was applied and these data were presented as medians and interquartile ranges. The qualitative data were presented as absolute and relative frequencies (Polit & Beck, 2017). The association between the nurses and physicians, including their attitudes and beliefs, was analysed with the chi‐square (*χ* ^2^) statistic for the qualitative variables at a 5% (*p* = .05) level of significance (Grove & Cipher, 2020).

### 3.6 Validity, reliability and rigour

When applying a cross‐cultural adaptation process to measure a phenomenon reported in the literature from other countries and cultures, researchers can either (a) develop a new instrument or (b) modify a previously validated instrument (Guillemin et al., 1993). Instruments to measure sexual health attitudes and beliefs of nurses and physicians in the context of primary care and in Spain were not available. As such, a systematic process was completed to transition the existing knowledge from the primarily English literature into a new instrument for application in Spain (Cha et al., 2007; Rattray & Jones, 2007).

The five steps to develop the questionnaire included the following: 1. literature review and variable abstraction, 2. variable definition and item construction, 3. item confirmation and domain review, 4. questionnaire evaluation and confirmation by experts (content validity) and 5. pilot test with small sample from the intended population (Escobar‐Pérez & Cuervo‐Martínez, 2008; Lynn, 1986). The SEX‐AP questionnaire was validated (V Aiken > 0.90) by 20 experts (Aiken, 1980, 1985; Beaton et al., 2000) and successfully pilot‐tested with 10 nurses and physicians from the target population; these data were not included in the analysis (Waltz et al., 2016). The study is reported in accordance with the Strengthening the Reporting of Observational Studies in Epidemiology (STROBE) statement (von Elm et al., 2007), with the completed checklist in File S3.

## 4 RESULTS

Completed questionnaires were submitted by 178 clinicians, including nurses (*N* = 85) and physicians (*N* = 93). Participants were mostly women (79%) with a median age of 45 years (interquartile range of 38–53 years). This sample represented 99% of the ideal sample size. The demographic data are presented in Table 1.

**TABLE 1:** Participant sociodemographic data

Variables	*n*	%
Sex		
Female	141	79.2
Male	37	20.8
Age^a^	45 [38–53]	
Occupation		
Nurse	85	47.8
Physician	93	51.2
Family and community specialist		
No	81	45.5
Yes	95	53.4
Prefer not to answer	2	1.1
Doctoral degree (PhD or ScD)		
No	164	92.1
Yes	7	3.9
In process	7	3.9
Master's degree		
No	110	61.8
Yes	64	36.0
In process	4	2.2
Postgraduate courses		
No	61	34.3
Yes	115	64.6
In process	2	1.1
Years of professional experience^a^	15 [8–21]	
Civil status		
Married	102	57.3
Single	54	30.3
Divorced	19	10.7
Prefer not to answer	3	1.7
Children		
No	66	37.1
Yes	112	62.9
Religion		
Catholic	77	43.3
Atheist	81	45.5
Other	6	3.4
Prefer not to answer	12	6.7
Importance of religion in life		
None or very little	111	62.4
Somewhat important	27	15.2
Important or very important	35	19.7
Prefer not to answer	5	2.8

Abbreviation: DK/DA, do not know/do not answer.

^a^ Median (ICR).

Nearly all participants (93.8%) reported patients ask them about sexual health concerns in a “few or some” of their visits. Not many participants (4.5%) reported asking patients about their sexual health in “all or almost all" of their visits. Most participants responded they were comfortable speaking with patients about sexual health, especially with women (69%; 124). But, only 8% of participants indicated they were uncomfortable addressing patient questions about sexual health concerns. Similarly, 84% of the participants “strongly disagreed” with feeling comfortable, compared with the 66% who felt safe working with the sexual health of patients. Regarding clinical management based on patient age, most participants (85%) “totally disagreed” with feeling uncomfortable about addressing sexual health concerns with young patients, while only 21% “totally agreed” with being uncomfortable talking about sexual health with older patients. In the comparative analysis of nurses and physician attitudes, statistically significant differences were observed about receiving questions from patients during visits (*p* = .020), as nurse more frequently reported receiving questions. Statistically significant differences were also found between male and female participants in feeling comfortable managing sexual health problems of men (*p* = .001). The complete data for the attitudes, item by profession, are presented in Table 2.

**TABLE 2:** Participant attitudes by profession and gender

Items and responses	*N* (%)	Physician (%)	Nurse (%)	*p*‐value	Female (%)	Male (%)	*p*‐value
Patients ask me about their sexual health…							
Somewhat or strongly disagree	11 (6.2)	9 (81.8)	2 (18.2)	.020*	7 (63.6)	4 (36.4)	.243
Somewhat or strongly agree	167 (93.8)	76 (45.5)	91 (54.5)		134 (80.2)	33 (19.8)	
I ask patients about their sexual health…							
Somewhat or strongly disagree	14 (7.9)	7 (50.0)	7 (50.0)	—	10 (71.4)	4 (28.6)	.679
Neither agree nor disagree	156 (87.7)	70 (44.9)	86 (55.1)		124 (79.5)	32 (20.5)	
Somewhat or strongly agree	8 (4.5)	8 (100.0)	0 (0.0)		7 (87.5)	1 (12.5)	
I feel comfortable discussing sexual health topics with male patients…							
Somewhat or strongly disagree	29 (16.3)	18 (62.1)	11 (37.9)	.124	28 (96.6)	1 (3.5)	.001*
Neither agree nor disagree	44 (24.7)	23 (52.3)	21 (47.7)		39 (88.6)	5 (11.4)	
Somewhat or strongly agree	105 (59.0)	44 (41.9)	61 (58.1)		74 (70.5)	31 (29.5)	
I feel comfortable discussing sexual health topics with female patients…							
Somewhat or strongly disagree	17 (9.6)	9 (52.9)	8 (47.1)	.836	15 (88.2)	2 (11.8)	.671
Neither agree nor disagree	38 (21.4)	19 (50.0)	19 (50.0)		29 (76.3)	9 (23.7)	
Somewhat or strongly agree	123 (69.1)	57 (46.3)	66 (53.7)		97 (78.9)	26 (21.1)	
I feel comfortable discussing sexual health topics with LGBTIQ patients…							
Somewhat or strongly disagree	30 (16.9)	15 (50.0)	15 (50.0)	.793	28 (93.3)	2 (6.7)	.089
Neither agree nor disagree	47 (26.4)	24 (51.1)	23 (48.9)		37 (78.7)	10 (21.3)	
Somewhat or strongly agree	101 (56.7)	46 (45.5)	55 (54.5)		76 (75.3)	25 (24.8)	
I do not like when patients ask me about their sexual health…							
Somewhat or strongly disagree	131 (73.6)	62 (47.3)	69 (52.7)	.847	103 (78.6)	28 (21.4)	.541
Neither agree nor disagree	33 (18.5)	17 (51.5)	16 (48.5)		28 (84.9)	5 (15.2)	
Somewhat or strongly agree	14 (7.9)	6 (42.9)	8 (57.1)		10 (71.4)	4 (28.6)	
I am professionally interested in sexual health topics in clinical practice…							
Somewhat or strongly disagree	12 (6.7)	5 (41.7)	7 (58.3)	.713	7 (58.3)	5 (41.7)	.149
Neither agree nor disagree	54 (30.3)	24 (44.4)	30 (55.6)		42 (77.8)	12 (22.2)	
Somewhat or strongly agree	112 (62.9)	56 (50.0)	56 (50.0)		92 (82.1)	20 (17.9)	
I feel comfortable discussing sexual health topics with patients…							
Somewhat or strongly disagree	150 (84.3)	72 (48.0)	78 (52.0)	.879	120 (80.0)	30 (20.0)	.549
Somewhat or strongly agree	28 (15.7)	13 (46.4)	15 (53.6)		21 (75.0)	7 (25.0)	
I feel confident discussing sexual health topics with patients…							
Somewhat or strongly disagree	60 (33.7)	33 (55.0)	27 (45.0)	.167	51 (85.0)	9 (15.0)	.175
Somewhat or strongly agree	118 (66.3)	52 (44.1)	66 (55.9)		90 (76.3)	28 (23.7)	
I feel comfortable discussing sexual health topics with young patients…							
Somewhat or strongly disagree	152 (85.4)	70 (46.1)	82 (54.0)	.272	119 (78.3)	33 (21.7)	.605
Somewhat or strongly agree	26 (14.6)	15 (57.7)	11 (42.3)		22 (84.6)	4 (15.4)	
I feel comfortable discussing sexual health issues with adult patients…							
Somewhat or strongly disagree	145 (81.5)	65 (44.8)	80 (55.2)	.155	112 (77.2)	33 (22.8)	—
Neither agree nor disagree	2 (1.1)	1 (50.0)	1 (50.0)		2 (100.0)	0 (0.0)	
Strongly or somewhat agree	31 (17.4)	19 (61.3)	12 (38.7)		27 (87.1)	4 (12.9)	
I feel comfortable discussing sexual health topics with elderly patients…							
Somewhat or strongly disagree	139 (78.1)	65 (46.8)	74 (53.2)	.855	108 (77.7)	31 (22.3)	—
Neither agree nor disagree	2 (1.1)	1 (50.0)	1 (50.0)		2 (100.0)	0 (0.0)	
Somewhat or strongly agree	37 (20.8)	19 (51.4)	18 (48.7)		31 (83.8)	6 (16.2)	

* Statistically significant median [ICR].

Regarding the beliefs of the participants, most (81%) reported indifferently that physicians and nurses are the right people to manage sexual health in primary care. Although almost half (46%) considered themselves appropriately prepared to manage issues, most participants (83%) reported they require more training to manage sexual health problems with greater confidence. In daily clinical practice, about a third of the participants (31%) believed sexual health is a priority; however, most participants (79%) lack the time to speak with patients about their sexual health concerns. Furthermore, many participants (63%) believe patients are not comfortable speaking with them about sexual health problems. The participants (64%) generally believed adolescents require more preventive education about sexual health than other age groups. Moreover, statistically significant differences were observed between nurses and physicians about needing more information to safely discuss sexual topics with patients (*p* = .025) and about having received enough training to address sexual health concerns (*p* = .008). In both cases, nurses reported having enough information and receiving the appropriate training. The complete results are presented in Table 3.

**TABLE 3:** Participant beliefs by profession and gender

Items and Responses	*N* (%)	Physician (%)	Nurse (%)	*p*‐value	Female (%)	Male (%)	*p*‐value
I believe the most appropriate health professional for patients to discuss sexual health topics with is/are…							
Family physician or nurse practitioner	144 (80.9)	62 (43.1)	82 (56.9)	—	116 (80.6)	28 (19.4)	—
Nurse practitioner	14 (7.9)	13 (92.9)	1 (7.1)		9 (64.3)	5 (35.7)	
Family physician	7 (3.9)	2 (28.6)	5 (71.4)		4 (57.2)	3 (42.9)	
Other physicians	4 (2.2)	3 (75.0)	1 (25.0)		4 (100.0)	0 (0.0)	
Other professionals	6 (3.4)	4 (66.7)	2 (33.3)		5 (83.3)	1 (16.7)	
None of the above	2 (1.1)	0 (0.0)	2 (100.0)		2 (100.0)	0 (0.0)	
Prefer not to answer or do not know	1 (0.6)	1 (100.0)	0 (0.0)		1 (100.0)	0 (0.0)	
I believe the age group most needing preventive sexual health education is…							
Childhood	3 (1.7)	1 (33.3)	2 (66.7)	–	2 (66.7)	1 (33.3)	–
Adolescence	113 (63.5)	54 (47.8)	59 (52.2)		89 (78.7)	24 (21.2)	
Adults	8 (4.5)	4 (50.0)	4 (50.0)		6 (75.0)	2 (25.0)	
Elders	53 (29.8)	26 (49.1)	27 (50.9)		43 (81.1)	10 (18.9)	
All of them	1 (0.6)	0 (0.0)	1 (100.0)		1 (100.0)	0 (0.0)	
I believe the age group least needing preventive sexual health education is…							
Childhood	25 (14.1)	14 (56.6)	11 (20.4)	—	21 (75.0)	4 (25.0)	—
Adolescence	2 (1.1)	1 (50.0)	1 (50.0)		1 (50.0)	1 (50.0)	
Adults	13 (7.3)	6 (46.2)	7 (53.9)		8 (61.5)	5 (38.5)	
Elderly	65 (36.5)	30 (46.2)	35 (53.9)		49 (75.4)	16 (24.6)	
All of them	18 (10.1)	12 (66.7)	6 (33.3)		15 (83.3)	3 (16.7)	
None of the above	47 (26.4)	20 (42.6)	27 (57.5)		40 (85.1)	7 (14.9)	
Prefer not to answer or do not know	8 (4.5)	2 (25.0)	6 (75.0)		7 (87.5)	1 (12.5)	
I believe I am appropriately trained to discuss sexual health topics with patients…							
Somewhat or strongly disagree	46 (25.8)	27 (58.7)	19 (41.3)	.226	39 (84.8)	7 (15.2)	.465
Neither agree nor disagree	50 (28.1)	22 (44.0)	28 (56.0)		40 (80.0)	10 (20.0)	
Somewhat or strongly agree	82 (46.1)	36 (43.9)	46 (56.1)		62 (75.6)	20 (24.4)	
I believe I should receive more training to confidently discuss sexual health topics with patients…							
Somewhat or strongly disagree	12 (6.7)	8 (66.7)	4 (33.3)	.025*	11 (91.7)	1 (8.3)	.543
Neither agree nor disagree	19 (10.7)	4 (21.1)	15 (79.0)		14 (73.7)	5 (26.3)	
Somewhat or strongly agree	147 (82.6)	73 (49.7)	74 (50.3)		116 (78.9)	31 (21.1)	
I believe I received sufficient university training to discuss sexual health topics with patients…							
Somewhat or strongly disagree	130 (73.0)	56 (43.1)	74 (56.9)	.008*	102 (78.5)	28 (21.5)	.872
Neither agree nor disagree	23 (12.9)	18 (78.3)	5 (21.8)		18 (78.3)	5 (21.7)	
Somewhat or strongly agree	25 (14.0)	11 (44.0)	14 (56.0)		21 (84.0)	4 (16.0)	
I believe sexual health is a priority in my clinical practice…							
Somewhat or strongly disagree	55 (30.9)	31 (56.4)	24 (43.6)	.210	43 (78.2)	12 (21.8)	.543
Neither agree nor disagree	87 (48.9)	36 (41.4)	51 (58.6)		67 (77.0)	20 (23.0)	
Somewhat or strongly agree	36 (20.2)	18 (50.0)	18 (50.0)		31 (86.1)	5 (13.9)	
I believe my colleagues discuss sexual health topics with their patients…							
Somewhat or strongly disagree	90 (50.6)	43 (47.8)	47 (52.2)	.695	70 (77.8)	20 (22.2)	.654
Neither agree nor disagree	44 (24.7)	23 (52.3)	21 (47.7)		34 (77.3)	10 (22.7)	
Somewhat or strongly agree	44 (24.7)	19 (43.2)	25 (56.8)		37 (84.1)	7 (15.9)	
I believe I do not have enough time to discuss sexual health topics with patients…							
Somewhat or strongly disagree	28 (15.7)	12 (42.9)	16 (57.1)	.365	21 (75.0)	7 (25.0)	.829
Neither agree nor disagree	10 (5.6)	7 (70.0)	3 (30.0)		8 (80.0)	2 (20.0)	
Somewhat or strongly agree	140 (78.7)	66 (47.1)	74 (52.9)		112 (80.0)	28 (20.0)	
I believe patients are not comfortable discussing sexual health topics with health professionals…							
Somewhat or strongly disagree	64 (36.0)	30 (46.9)	34 (53.1)	.938	49 (76.6)	15 (23.4)	.343
Neither agree nor disagree	2 (1.1)	1 (50.0)	1 (50.0)		1 (50.0)	1 (50.0)	
Somewhat or strongly agree	112 (62.9)	54 (48.2)	58 (51.8)		91 (81.3)	21 (18.8)	
In clinical practice, I believe there are higher priority needs to discuss rather than sexual health topics…							
Somewhat or strongly disagree	82 (46.1)	45 (54.9)	37 (45.1)	.199	65 (79.3)	17 (20.7)	1.000
Neither agree nor disagree	21 (11.8)	8 (38.1)	13 (61.9)		17 (81.0)	4 (19.0)	
Somewhat or strongly agree	75 (42.1)	32 (42.7)	43 (57.3)		59 (78.7)	16 (21.3)	

* Statistically significant median [ICR].

## 5 DISCUSSION

This study indicates most participants working in primary care centres believe patients seldomly ask them about sexual health as they are not comfortable speaking about sex. Consequently, most participants in this study do not ask their patients about their sexual health. Similarly, a study from Denver (USA) reported almost two in three primary care physicians never or almost never addressed sexual health topics with their patients (Bober et al., 2009). The same authors also found most participants consider physicians and family nurse practitioners to be the right health professionalto address sexual health issues with patients. Contrary results have been reported by other studies as participants were uneasy or not clear about the right health professional responsible for initiating conversations about sexual health with patients (Hordern & Street, 2007; Stead et al., 2003).

In this study, about half the participants considered sexual health to be a lower level patient priority compared with other health areas, indicating in most cases they lacked the time to speak about sexual topics with patients. This finding evidences the challenges primary care professionals encounter as they are managing too many patients during extended working hours with shorter visits, resulting in little time to address sexual health (Stead et al., 2001; Wiggins et al., 2007). This seems to be similar to the problems encountered by nurses working in hospital settings, as they reported not being inclined to spend time discussing sexual health issues with their patients (Arikan et al., 2015). Although the present study found participants believed adolescents require more preventive sexual health education, similar studies, although old and in different contexts, indicate conversations about sexual health between physicians and adolescents rarely occur (Alexander et al., 2014; Boekeloo, 2014; Maheux et al., 1995; O'Keeffe & Clarke‐Pearson, 2011; Schuster et al., 1996). When the conversations do occur, they are on average 36 s (Alexander et al., 2014) and focused on learning if the adolescents were sexually active (Fuzzell et al., 2016). Similar findings have been reported in the two other studies from Spain (Dalfó‐Pibernat et al., 2015; Raya‐Tena et al., 2019).

Previous studies also identified characteristics such as age, gender, race and sexual orientation can discourage clinicians from discussing sexual health issues with their patients (Bober et al., 2009; Stead et al., 2003). In this study, almost all participants reported not having concerns about managing sexual health issues with younger patients, compared with most participants being uncomfortable discussing sex with older patients and other participants feeling uncomfortable with LGTBI patients. Despite the geographical and cultural differences, there is some resistance on the part of health professionals about discussing sexual health issues.

Although nurses consider sexual conversations to be private and intimate (Arikan et al., 2015), this study found half the participants are confident enough and willing to engage patients in discussions about sex. Generally, physicians felt insecure about how to discuss sexual health, especially sexual orientation, with younger patients recognizing their ignorance about the availability of community resources (Lena et al., 2002). In this regard, nurses consider speaking to patients about their sexual health is an important responsibility (Yip et al., 2015). Nurses considering this important feel more confident about their ability to handle issues and invite patients to talk about sex (Arikan et al., 2015; Magnan & Reynolds, 2006). As most physicians reported their sexual health knowledge was limited, they would like more education to manage the issue safely and effectively.

This study found a difference between nurses and physicians about their comfort in engaging patients in sexual health conversations. For example, more often than physicians, the nurses in this study felt safer discussing sexual matters with patients, reported more sufficient knowledge to address questions, and believed they can effectively manage issues with either men or women. A systematic review of sexuality in health care determined sexual health care is not viewed by physicians as a medical problem (Fennell & Grant, 2019). Similarly, in addressing sexual health, Klaeson et al. (2017) noted the medical paradigm needs to shift to person‐centred care to address sexual health with a more holistic perspective similar to nurses. As medical curriculums are not focused on holistic caring, they are missing education and training focused on important concepts such as empathy, compassion, and communication (Ha & Longnecker, 2010; Patel et al., 2019), including a lack of instruction for psychosocial concerns such as sexual health (Dennis & Elstein, 1980; Wagner, 2005).

Physician comfort with managing psychosocial concerns has been correlated to speaking with patients about their sexual health (Tsimtsiou et al., 2006). Previous physician training in communication was the strongest predictor for addressing sexual health. Grounded in a human caring ontology (Watson, 2009), nursing curriculum emphasizes altruism, authentic presence, communication, and therapeutic care (Landers et al., 2014). For this reason, nurses are probably most capable of shifting the current medical paradigm to welcome sexual health as a normal primary care conversation.

Finally, sexual health has long been noted to be an educational priority for health science programmes, residency training, and practicing clinicians (Ford et al., 2013). Although clinicians are required to complete continuing education to maintain licensure in many countries (Institute of Medicine, 2010; Karas et al., 2020; Sherwood & Shaffer, 2014), there are limited intervention studies in this area (Clark et al., 2012; Wazqar, 2020). Online continuing sexual health education (Bos‐Bonnie et al., 2017; Win et al., 2015) tailored for clinicians in a modular approach (Case beer et al., 2002) has been reported to increase knowledge and change clinical practice (Guy et al., 2011; Shabsigh et al., 2009). In this regard, clinicians can access free and low‐cost sexual health courses, two to six hours, or enroll in postgraduate certificate programs which are longer and more expensive. Several organizations provide current clinical practice resources for sexual health including links for external continuing education and university‐based programmes (Centers for Disease Control & Prevention, 2017, 2018, 2019; Royal College of Nursing, 2020; World Association for Sexual Health, 2020; World Organization of Family Doctors, 2019).

### 5.1 Limitations

Despite incorporating strategies to reduce bias and increase generalizability (Malone et al., 2014), there are five study limitations that need to be discussed. First, response and information bias are possible when using a questionnaire (Choi & Pak, 2005). To minimize this bias, the questionnaire was anonymous and sent through an external web link (Keough & Tanabe, 2011). Second, despite the large number of sampling locations, selection bias needs to be considered since the participants responding to the survey might have been more familiar or motivated by the topic. Third, the non‐probabilistic sampling can have an impact on the results (Polit & Beck, 2017). However, the response rate for the present study was higher (Gök & Demir Korkmaz, 2018) than the previously cited studies. Fourth, the questionnaire could have increased social desirability bias, as participants respond how they believed would be favourably viewed by researchers (Waltz et al., 2016). This is not likely as there were multiple responses contrary to a favourable view. Fifth, the sample may be subject to participation biases due to non‐response, but participation was high and the item summary is disclosed in the tables (Badger & Werrett, 2005). Despite these five anticipated limitations, the study was designed to minimize the potential for errors and biases. Finally, this is one of the first sexual health studies comparing nurses and physicians with a representative sample from multiple locations.

## 6 CONCLUSION

This study found primary care nurses and physicians in Barcelona (Spain) do not generally feel comfortable speaking with their patients about sexual health. They avoid asking questions about sexual health despite recognizing their important role in helping patients achieve satisfaction with their sexual health. However, nurses are more willing, comfortable, and capable of engaging their patients in sexual health discussions than their physician colleagues. Clearly, the work environment and self‐reported knowledge deficits about addressing sexual health are the principle barriers to engaging patients in sexual discussions. Qualitative research may be helpful to unpacking the way participant attitudes and beliefs manifest as barriers or facilitators to discussing sexual health with patients. Applied nursing research is also necessary to translate the current evidence into strategies to guide clinical practice and to inform nursing and medical curriculums. Sexual health knowledge needs to be improved and communication skills further developed through additional training to stimulate more intentional discussions with patients. Other intervention strategies focused on promoting person‐centred care should be considered to help patients initiate the conversations with clinicians about their sexual health in a constructive and comfortable manner.

## CONFLICT OF INTEREST

No conflict of interest has been declared by the authors.

## AUTHOR CONTRIBUTIONS

AD‐P, GG‐V, JML‐M, MA‐G, NG‐G and PAP: Conception and design of the study; acquisition of data; analysis and interpretation of data; preparation of draft; critical revision for important intellectual content; final approval of the version to be published; and accountable for all aspects of the work in ensuring that questions related to the accuracy or integrity of any part of the work are appropriately investigated and resolved.

## Supporting information

File S1

File S2

File S3

## Data Availability

The data that support the findings of this study are available from the corresponding author upon reasonable request.
