# Supplementary material for: Attitudes and beliefs of nurses and physicians about managing sexual health in primary care: A multi‐site cross‐sectional comparative study
Source: Nurs Open. 2020 Oct 20;8(1):404–14. doi: 10.1002/nop2.641 (PMC7729806; doi:10.1002/nop2.641)
Supplement: Supplementary file 1 — File S1 [file NOP2-8-404-s001.docx]

Supplemental File 1. Sexualidad en Atención Primaria (SEX-AP) Cuestionario

Responda las siguientes preguntas honestamente y recuerde que no hay respuestas correctas o incorrectas.

| 1. Sexo… | a) Hombre  b) Mujer |
| --- | --- |
| 1. Edad (en años)… |  |
| 1. Ocupación… | a) Enfermera  b) Médico  c) No sabe / No contesta |
| 1. Especialidad familiar y comunitaria… | a) No  b) Si  c) No sabe / No contesta |
| 5. Doctorado… | a) No  b) Si  c) En curso |
| 6. Maestría… | a) No  b) Si  c) En curso |
| 7. Cursos de posgrado… | a) No  b) Si  c) En curso |
| 1. Años de experiencia profesional… |  |
| 1. Estado civil… | a) Casado/a  b) Soltero/a  c) Divorciado/a  d) No sabe / No contesta |
| 1. ¿Tiene hijos? | a) No  b) Si |
| 11. Religión… | a) Católico/a  b) Ateo/a  c) Otro  d) No sabe / No contesta |
| 12. Importancia de la religión en la vida… | a) Nada  b) Muy poco  c) Algo importante  d) Importante  e) Muy importante  f) No sabe / No contesta |
| 13. Creo que el profesional más apropiado para discutir temas de salud sexual es… | a) Médico de familia  b) Enfermera de familia  c) Médico o enfermera de familia indistintamente  d) Otros médicos  e) Otros profesionales  f) Ninguna de las anteriores  h) No sabe / No contesta |
| 14. Creo que el grupo de edad que más necesita educación preventiva sobre salud sexual es… | a) Infancia  b) Adolescencia  c) Adultos  d) Ancianos  e) Todos ellos  f) Ninguna de las anteriores  g) No sabe / No contesta |
| 15. Creo que el grupo de edad que menos necesita educación preventiva sobre salud sexual es… | a) Infancia  b) Adolescencia  c) Adultos  d) Ancianos  e) Todos ellos  f) Ninguna de las anteriores  g) No sabe / No contesta |
| 16. Los/las pacientes me preguntan sobre su salud sexual… | a) Totalmente en desacuerdo  b) Algo en desacuerdo  c) Ni de acuerdo ni en desacuerdo  d) Algo de acuerdo  e) Totalmente de acuerdo |
| 17. Pregunto a mis pacientes sobre su salud sexual | a) Totalmente en desacuerdo  b) Algo en desacuerdo  c) Ni de acuerdo ni en desacuerdo  d) Algo de acuerdo  e) Totalmente de acuerdo |
| 18. Creo que estoy capacitado/a para hablar sobre salud sexual con los/las pacientes… | a) Totalmente en desacuerdo  b) Algo en desacuerdo  c) Ni de acuerdo ni en desacuerdo  d) Algo de acuerdo  e) Totalmente de acuerdo |
| 19. Creo que debería recibir más capacitación para hablar con seguridad sobre la salud sexual con los/las pacientes… | a) Totalmente en desacuerdo  b) Algo en desacuerdo  c) Ni de acuerdo ni en desacuerdo  d) Algo de acuerdo  e) Totalmente de acuerdo |
| 20. Me siento cómodo/a hablando de salud sexual con pacientes de sexo masculino… | a) Totalmente en desacuerdo  b) Algo en desacuerdo  c) Ni de acuerdo ni en desacuerdo  d) Algo de acuerdo  e) Totalmente de acuerdo |
| 21. Me siento cómodo/a hablando de salud sexual con pacientes de sexo femenino… | a) Totalmente en desacuerdo  b) Algo en desacuerdo  c) Ni de acuerdo ni en desacuerdo  d) Algo de acuerdo  e) Totalmente de acuerdo |
| 22. Me siento cómodo/a hablando de salud sexual con pacientes LGBTIQ*…  * LGBTIQ = lesbiana, gay, bisexual, transgénero, transexual, travesti, intersexual y queer | a) Totalmente en desacuerdo  b) Algo en desacuerdo  c) Ni de acuerdo ni en desacuerdo  d) Algo de acuerdo  e) Totalmente de acuerdo |
| 23. Creo que obtuve suficiente formación en la universidad para hablar sobre salud sexual con los/las pacientes… | a) Totalmente en desacuerdo  b) Algo en desacuerdo  c) Ni de acuerdo ni en desacuerdo  d) Algo de acuerdo  e) Totalmente de acuerdo |
| 24. Creo que los problemas de salud sexual son una prioridad en mi práctica clínica… | a) Totalmente en desacuerdo  b) Algo en desacuerdo  c) Ni de acuerdo ni en desacuerdo  d) Algo de acuerdo  e) Totalmente de acuerdo |
| 25. No me gusta cuando los/las pacientes me preguntan sobre temas de salud sexual… | a) Totalmente en desacuerdo  b) Algo en desacuerdo  c) Ni de acuerdo ni en desacuerdo  d) Algo de acuerdo  e) Totalmente de acuerdo |
| 26. Estoy profesionalmente interesado/a en la salud sexual… | a) Totalmente en desacuerdo  b) Algo en desacuerdo  c) Ni de acuerdo ni en desacuerdo  d) Algo de acuerdo  e) Totalmente de acuerdo |
| 27. Me siento cómodo/a discutiendo problemas de salud sexual con los/las pacientes… | a) Totalmente en desacuerdo  b) Algo en desacuerdo  c) Ni de acuerdo ni en desacuerdo  d) Algo de acuerdo  e) Totalmente de acuerdo |
| 28. Me siento seguro/a discutiendo asuntos sexuales con los/las pacientes… | a) Totalmente en desacuerdo  b) Algo en desacuerdo  c) Ni de acuerdo ni en desacuerdo  d) Algo de acuerdo  e) Totalmente de acuerdo |
| 29. Me siento cómodo/a hablando de salud sexual con pacientes jóvenes… | a) Totalmente en desacuerdo  b) Algo en desacuerdo  c) Ni de acuerdo ni en desacuerdo  d) Algo de acuerdo  e) Totalmente de acuerdo |
| 30. Me siento cómodo/a hablando de salud sexual con pacientes adultos… | a) Totalmente en desacuerdo  b) Algo en desacuerdo  c) Ni de acuerdo ni en desacuerdo  d) Algo de acuerdo  e) Totalmente de acuerdo |
| 31. Me siento cómodo/a hablando de salud sexual con pacientes de edad avanzada… | a) Totalmente en desacuerdo  b) Algo en desacuerdo  c) Ni de acuerdo ni en desacuerdo  d) Algo de acuerdo  e) Totalmente de acuerdo |
| 32. Creo que mis colegas discuten la salud sexual con sus pacientes… | a) Totalmente en desacuerdo  b) Algo en desacuerdo  c) Ni de acuerdo ni en desacuerdo  d) Algo de acuerdo  e) Totalmente de acuerdo |
| 33. Creo que no tengo tiempo para hablar sobre salud sexual con los/las pacientes… | a) Totalmente en desacuerdo  b) Algo en desacuerdo  c) Ni de acuerdo ni en desacuerdo  d) Algo de acuerdo  e) Totalmente de acuerdo |
| 34. Creo que los/las pacientes no se sienten cómodos/as hablando sobre salud sexual con los/las profesionales sanitarios… | a) Totalmente en desacuerdo  b) Algo en desacuerdo  c) Ni de acuerdo ni en desacuerdo  d) Algo de acuerdo  e) Totalmente de acuerdo |
| 35. Creo que en la práctica clínica hay intervenciones prioritarias que la salud sexual… | a) Totalmente en desacuerdo  b) Algo en desacuerdo  c) Ni de acuerdo ni en desacuerdo  d) Algo de acuerdo  e) Totalmente de acuerdo |
