# Supplementary material for: Attitudes and beliefs of nurses and physicians about managing sexual health in primary care: A multi‐site cross‐sectional comparative study
Source: Nurs Open. 2020 Oct 20;8(1):404–14. doi: 10.1002/nop2.641 (PMC7729806; doi:10.1002/nop2.641)
Supplement: Supplementary file 2 — File S2 [file NOP2-8-404-s002.docx]

Supplemental File 2. Sexual Health in Primary Care (SEX-PC) Questionnaire

Please answer the following questions honestly. Remember, there are no right or wrong responses to the questions

| 1. Your sex is… | 1. Male 2. Female |
| --- | --- |
| 1. Your age (in years) is… |  |
| 1. Your occupation is… | 1. Nurse 2. Physician 3. Prefer not to answer |
| 1. Are you specialized in family and/or community care… | 1. No 2. Yes 3. Prefer not to answer |
| 5. Do you have a doctoral degree (other than medical degree)… | 1. No 2. Yes 3. On-going |
| 6. Do you have a master’s degree… | 1. No 2. Yes 3. On-going |
| 7. Do you have postgraduate courses… | 1. No 2. Yes 3. On-going |
| 1. Your years of professional experience… |  |
| 1. Your civil status is… | 1. Married 2. Single 3. Divorced 4. Prefer not to answer |
| 1. Do you have children… | 1. No 2. Yes |
| 11. Your religion is… | 1. Catholic 2. Atheist 3. Other 4. Prefer not to answer |
| 12. The importance of religion in your life is… | 1. Not at all 2. Very little 3. Somewhat important 4. Important 5. Very important 6. Prefer not to answer |
| 13. I believe the most appropriate health professional for patients to discuss sexual health topics with is/are… | 1. Family physician 2. Family nurse 3. Family physician and/or nurse 4. Other physician(s) 5. Other professional(s) 6. None of the above 7. All the above 8. Prefer not to answer |
| 14. I believe the age group most needing preventive sexual health education is… | 1. Children 2. Adolescent 3. Adults 4. Elderly 5. All the above 6. None of the above 7. Prefer not to answer |
| 15. I believe the age group least needing preventive sexual health education is… | 1. Childhood 2. Adolescence 3. Adults 4. Elders 5. All of them 6. None of the above 7. Prefer not to answer |
| 16. Patients ask me about their sexual health… | 1. Strongly disagree 2. Somewhat disagree 3. Neither agree nor disagree 4. Somewhat agree 5. Strongly agree |
| 17. I ask to my patients about their sexual health… | 1. Strongly disagree 2. Somewhat disagree 3. Neither agree nor disagree 4. Somewhat agree 5. Strongly agree |
| 18. I believe I am appropriately trained to discuss sexual health topics with patients… | 1. Strongly disagree 2. Somewhat disagree 3. Neither agree nor disagree 4. Somewhat agree 5. Strongly agree |
| 19. I believe I should receive more training to competently discuss sexual health topics with patients… | 1. Strongly disagree 2. Somewhat disagree 3. Neither agree nor disagree 4. Somewhat agree 5. Strongly agree |
| 20. I feel comfortable discussing sexual health topics with male patients… | 1. Strongly disagree 2. Somewhat disagree 3. Neither agree nor disagree 4. Somewhat agree 5. Strongly agree |
| 21. I feel comfortable discussing sexual health topics with female patients… | 1. Strongly disagree 2. Somewhat disagree 3. Neither agree nor disagree 4. Somewhat agree 5. Strongly agree |
| 22. I feel comfortable discussing sexual health topics with LGBTIQ* patients…  *The term LGBTIQ refers to lesbian, gay, bisexual, transgender/transsexual, intersex and/or queer. | 1. Strongly disagree 2. Somewhat disagree 3. Neither agree nor disagree 4. Somewhat agree 5. Strongly agree |
| 23. I believe I received sufficient training in my university program to discuss sexual health topics with patients… | 1. Strongly disagree 2. Somewhat disagree 3. Neither agree nor disagree 4. Somewhat agree 5. Strongly agree |
| 24. I believe sexual health is a priority in my clinical practice… | 1. Strongly disagree 2. Somewhat disagree 3. Neither agree nor disagree 4. Somewhat agree 5. Strongly agree |
| 25. I do not like when patients ask me about their sexual health… | 1. Strongly disagree 2. Somewhat disagree 3. Neither agree nor disagree 4. Somewhat agree 5. Strongly agree |
| 26. I am professionally interested in sexual health topics in clinical practice… | 1. Strongly disagree 2. Somewhat disagree 3. Neither agree nor disagree 4. Somewhat agree 5. Strongly agree |
| 27. I feel comfortable discussing sexual health topics with patients… | 1. Strongly disagree 2. Somewhat disagree 3. Neither agree nor disagree 4. Somewhat agree 5. Strongly agree |
| 28. I feel confident discussing sexual health topics with patients… | 1. Strongly disagree 2. Somewhat disagree 3. Neither agree nor disagree 4. Somewhat agree 5. Strongly agree |
| 29. I feel comfortable discussing sexual health topics with young patients… | 1. Strongly disagree 2. Somewhat disagree 3. Neither agree nor disagree 4. Somewhat agree 5. Strongly agree |
| 30. I feel comfortable discussing sexual health issues with adult patients… | 1. Strongly disagree 2. Somewhat disagree 3. Neither agree nor disagree 4. Somewhat agree 5. Strongly agree |
| 31. I feel comfortable discussing sexual health topics with elderly patients | 1. Strongly disagree 2. Somewhat disagree 3. Neither agree nor disagree 4. Somewhat agree 5. Strongly agree |
| 32. I believe my colleagues discuss sexual health topics with their patients… | 1. Strongly disagree 2. Somewhat disagree 3. Neither agree nor disagree 4. Somewhat agree 5. Strongly agree |
| 33. I believe I do not have enough time to discuss sexual health topics with patients… | 1. Strongly disagree 2. Somewhat disagree 3. Neither agree nor disagree 4. Somewhat agree 5. Strongly agree |
| 34. I believe patients are not comfortable discussing sexual health topics with health professionals… | 1. Strongly disagree 2. Somewhat disagree 3. Neither agree nor disagree 4. Somewhat agree 5. Somewhat agree |
| 35. In clinical practice, I believe there are other higher priority needs to discuss rather than sexual health topics … | 1. Strongly disagree 2. Somewhat disagree 3. Neither agree nor disagree 4. Somewhat agree 5. Strongly agree |
